# Supplementary figures and images for: Reversing chemorefraction in colorectal cancer cells by controlling mucin secretion
Source: eLife. 2022 Feb 8;11:e73926. doi: 10.7554/eLife.73926 (PMC8846583; doi:10.7554/eLife.73926)

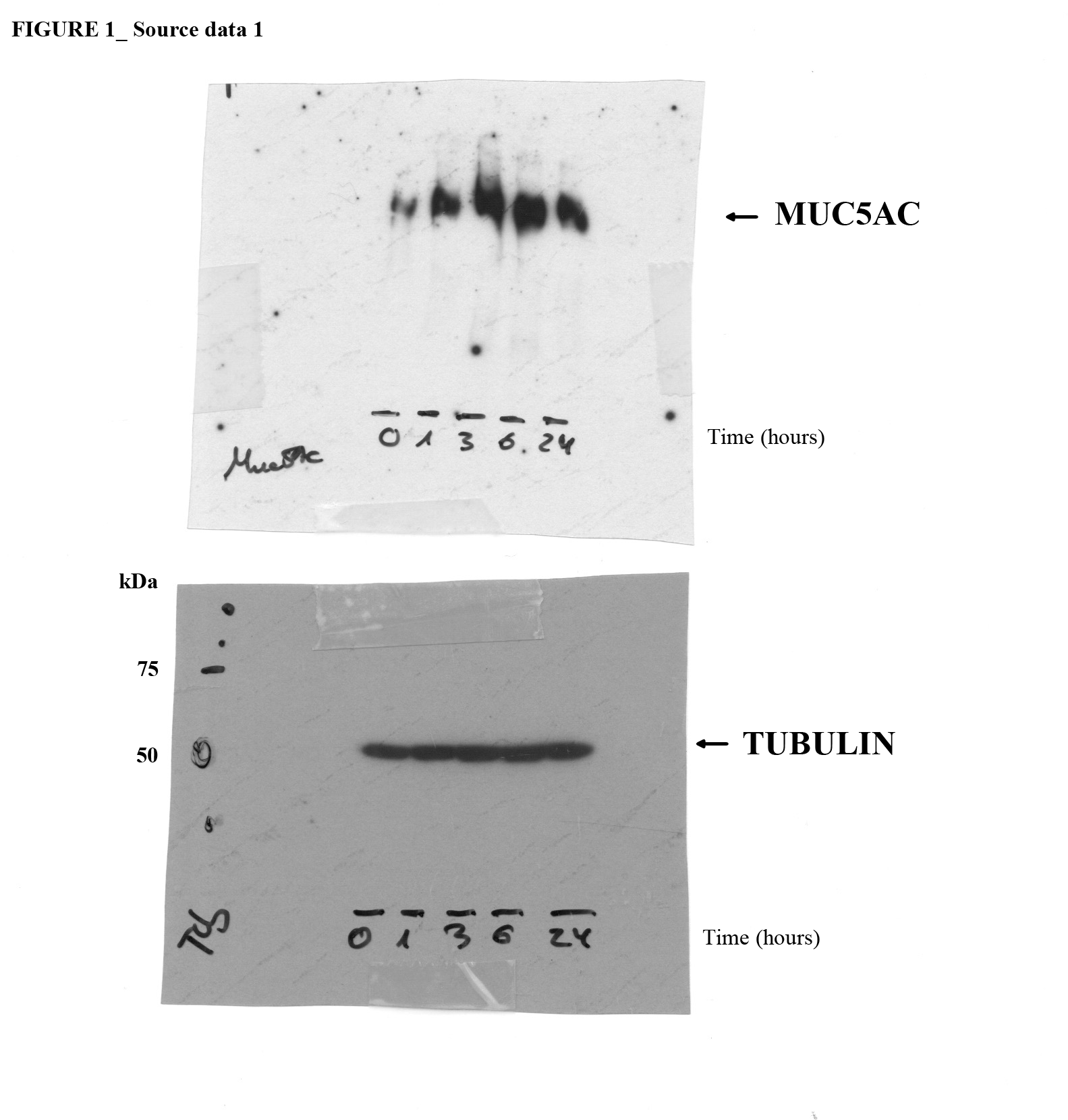

Supplement: Figure 1—source data 1. [file elife-73926-fig1-data1.zip › f4fbffbc-d8bd-4764-976c-288990d56d5d.tif]

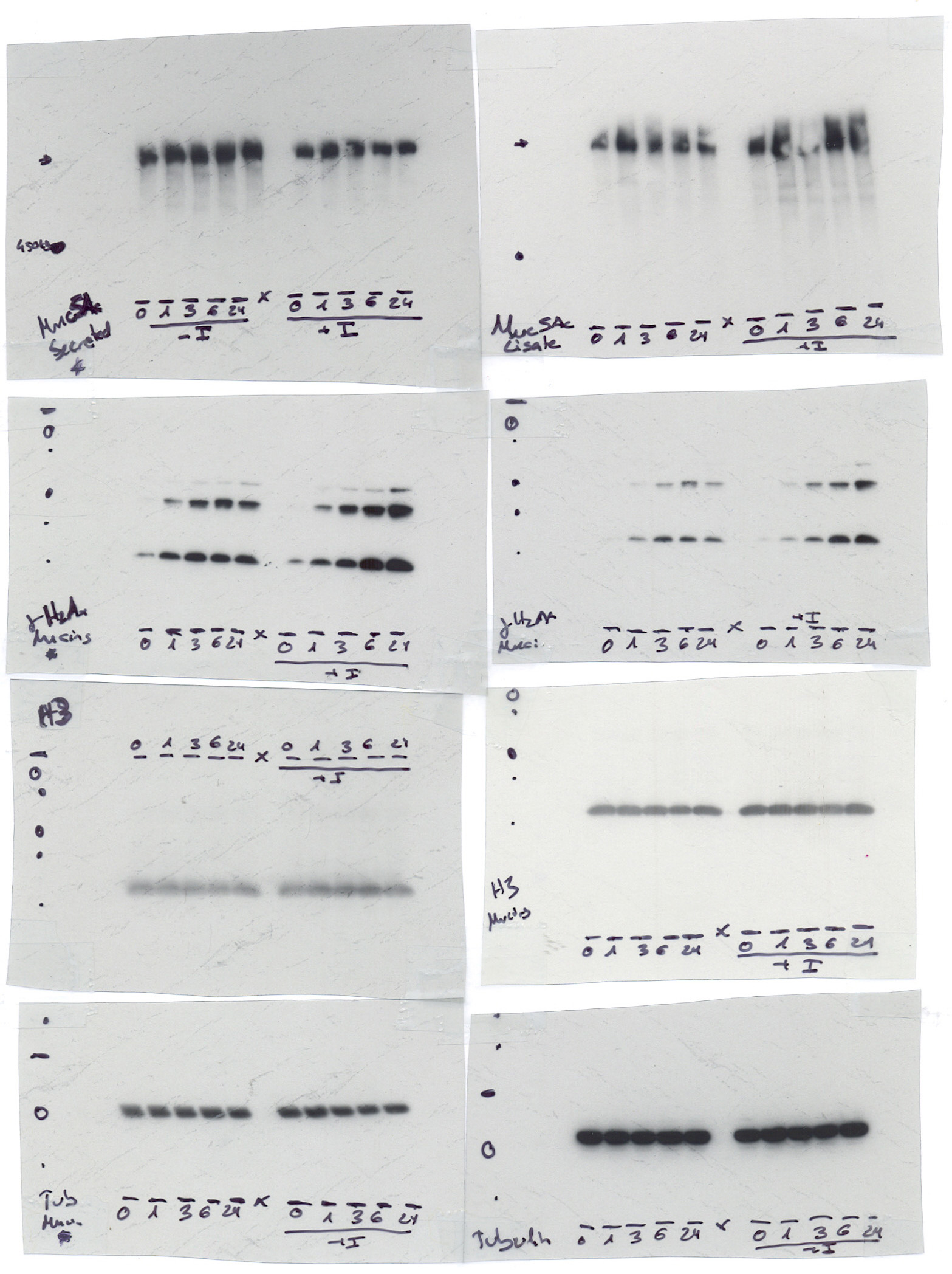

Supplement: Figure 4—source data 1. [file elife-73926-fig4-data1.zip › Source data_Figure 4.jpg]
